# Anti-methanogenic potential of seaweeds and seaweed-derived compounds in ruminant feed: current perspectives, risks and future prospects

**DOI:** 10.1186/s40104-023-00946-w

**Published:** 2023-12-02

**Authors:** Ailbhe McGurrin, Julie Maguire, Brijesh K. Tiwari, Marco Garcia-Vaquero

**Affiliations:** 1 Section of Food and Nutrition, School of Agriculture and Food Science, University College Dublin, Dublin 4, Belfield, Ireland; 2 TEAGASC, Food Research Centre, Dublin 15, Ashtown, Ireland; 3 Bantry Marine Research Station Ltd, Bantry, Co. Cork, P75 AX07, Gearhies, Ireland

**Keywords:** Asparagopsis, Bromoform, Methanogenesis, Phlorotannin, Saponin, Tannin

## Abstract

With methane emissions from ruminant agriculture contributing 17% of total methane emissions worldwide, there is increasing urgency to develop strategies to reduce greenhouse gas emissions in this sector. One of the proposed strategies is ruminant feed intervention studies focused on the inclusion of anti-methanogenic compounds which are those capable of interacting with the rumen microbiome, reducing the capacity of ruminal microorganisms to produce methane. Recently, seaweeds have been investigated for their ability to reduce methane in ruminants in vitro and in vivo, with the greatest methane abatement reported when using the red seaweed *Asparagopsis taxiformis* (attributed to the bromoform content of this species). From the literature analysis in this study, levels of up to 99% reduction in ruminant methane emissions have been reported from inclusion of this seaweed in animal feed, although further in vivo and microbiome studies are required to confirm these results as other reports showed no effect on methane emission resulting from the inclusion of seaweed to basal feed. This review explores the current state of research aiming to integrate seaweeds as anti-methanogenic feed additives, as well as examining the specific bioactive compounds within seaweeds that are likely to be related to these effects. The effects of the inclusion of seaweeds on the ruminal microbiome are also reviewed, as well as the future challenges when considering the large-scale inclusion of seaweeds into ruminant diets as anti-methanogenic agents.

## Introduction

It is now abundantly clear that anthropogenic activities have caused increased greenhouse gas (GHG) emissions and the current climate crisis [1, 2]. The final instalment of the Intergovernmental Panel on Climate Change (IPCC) 6^th^ Assessment Report stated that global surface temperatures reached 1.1 °C above pre-industrial levels between 2011 and 2020 [3], and that unless there are immediate and substantial reductions to GHG emissions worldwide, global warming will rise beyond the 1.5–2 °C threshold in the next 20 years [4]. While the GHG methane (CH_4_) has a shorter half-life (8.4 years) than CO_2_, the world’s largest GHG contributor, it has a global warming potential (GWP) 28–34 times higher than CO_2_ [5]. CH_4_ emissions from ruminant farming contribute 39% of all agricultural CH_4_ emissions and about 17% of total CH_4_ emissions worldwide [6, 7]. These CH_4_ emissions are projected to be a bottleneck for mitigation of GHG in future years; with 40%–78% of global CH_4_ emissions predicted to be as a result of ruminant farming by 2100 [8]. Various mitigation strategies have been proposed and implemented to date directly and indirectly targeting CH_4_ emissions, including an increasingly crucial global shift towards plant-based diets worldwide. Due to its comparatively short half-life, targeted CH_4_ reduction strategies have been suggested as the impact of these may be measured in the relatively short term [9]. Such targeted CH_4_ reduction strategies include a variety of anti-methanogenic feed additives, such as chemically synthesised compounds and plant secondary metabolites, targeted CH_4_ inhibitors administered to ruminants, and vaccinations [6]. These strategies can differ in their potential to reduce CH_4_ and, to ensure successful adoption in agriculture, must not negatively impact overall animal health or performance. The main strategies currently available to mitigate CH_4_ emissions are summarised by Kumar et al. [6].

### Seaweeds as anti-methanogenic agents

Among the most achievable interventions to attempt to mitigate CH_4_ emissions are feed additives with anti-methanogenic activity. These interventions are easy to integrate into existing agricultural practices and, depending on the particular feed additive, do not present as much regulatory challenges as administering anti-methanogenic agents separately to feed [10]. Plant secondary metabolites such as tannins, saponins, flavonoids, and chemical compounds such as 3-nitrooxypropanol (3-NOP) and ethyl-3-NOP, have been explored as feed additives. All such feed additives inhibit methanogenesis in some way, either via their direct biochemical activity (e.g., 3-NOP binds competitively to enzymes necessary for methanogenesis) or by the indirect manipulation of the ruminal microbiome (tannins have been reported to decrease H_2_ production due to reductions in fibre digestion) [11].

Incorporating seaweeds (macroalgae) as an anti-methanogenic feed additive has been investigated and has gained research interest in recent years [7, 12–14]. However, these strategies have reported variable results. Some in vitro studies report > 95% CH_4_ inhibition by using seaweed as a feed additive [15–17], while other studies report no inhibition [18, 19]. The most promising seaweed species currently researched to reduce CH_4_ emissions include *Asparagopsis taxiformi*s and *Ascophyllum nodosum*, generally attributed to their contents of halogenated compounds and phlorotannins, respectively [20]. Seaweeds have been used as a livestock feed for millennia, mainly in coastal communities, to provide nutritive value to animals [21]. Orpin et al. [22] determined that the sheep of the remote island of North Ronaldsay (Orkney, United Kingdom) whose diet consists of > 90% seaweed, had a different microbiome community to standard pasture fed sheep, including decreased levels of cellulolytic bacteria. Since then the incorporation of seaweeds as an anti-methanogenic feed additive has been increasingly explored, aiming to reduce global anthropogenic GHG emissions, as well as providing nutrient value to livestock including protein and polyunsaturated fatty acids (PUFAs) [23], and contributing to marine carbon sequestration if the biomass is cultivated offshore. Seaweeds can also accumulate minerals, such as iodine and bromine amongst others, which can be toxic to human health when reaching certain thresholds and thus, they must be carefully monitored in the animal products of ruminants fed seaweed [24, 25]. Moreover, one of the main bioactive compounds attributed to the anti-methanogenic effect of seaweeds, bromoform, has also been associated with human health concerns and it has been reported to be an ozone-depleting compound [26, 27]. Therefore, this review aims to explore the potential of seaweed to reduce CH_4_ emissions in ruminants, as well as to examine the particular compounds responsible for these effects and their overall impacts on the rumen microbiome. The concerns or monitoring recommendations reported currently by researchers will also be detailed aiming to provide a comprehensive view of the current and future scenario of the widespread use of these compounds.

### Methanogenesis and the rumen microbiome

**Fig. 1:**
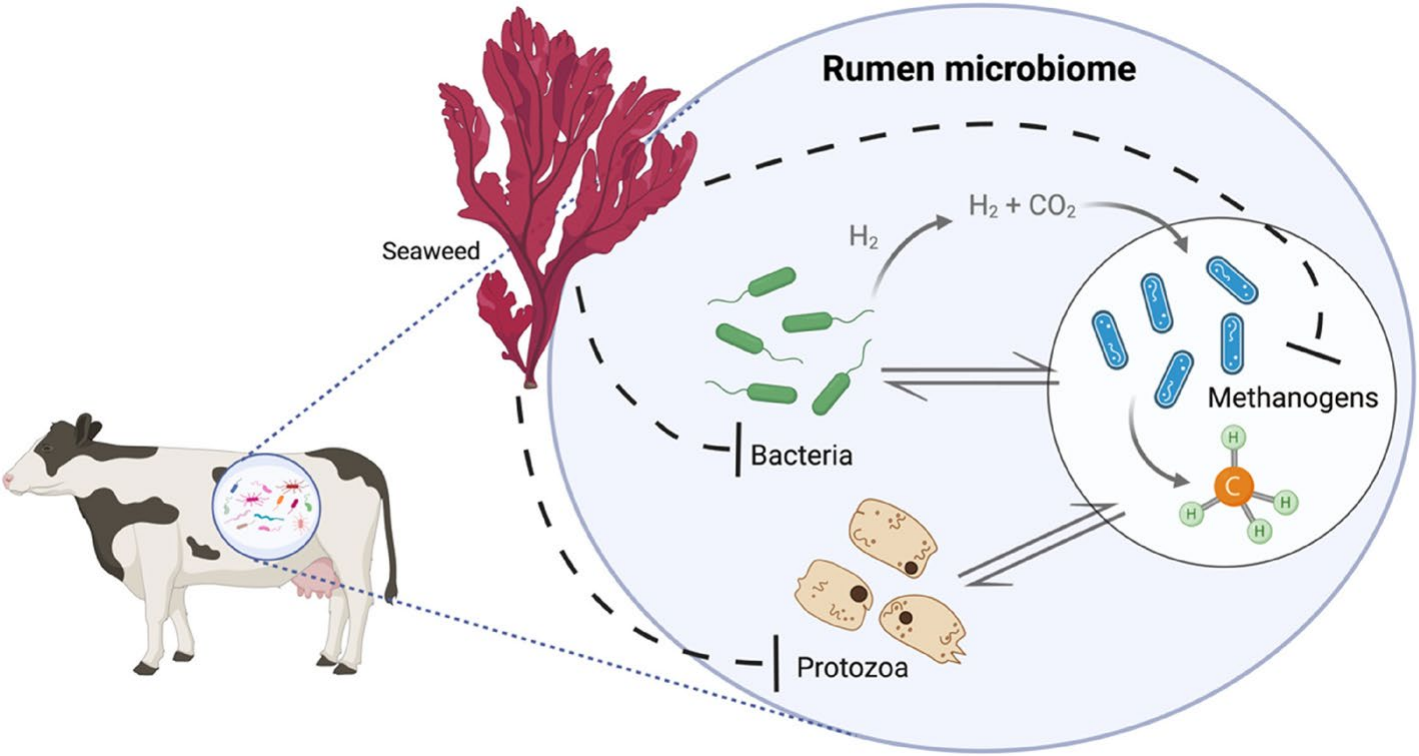
Representation of seaweed impact on the rumen microbiome. Seaweed and associated bioactive compounds have been reported to act on methanogens, bacteria, and protozoa in the rumen microbiome, thus either directly or indirectly reducing amount of CH_4_ produced. Created with BioRender.com

The rumen contains a complex microbiome of bacteria, archaea, viruses, fungi, and protozoa which degrade and ferment cellulosic material, resulting in the production of volatile fatty acids (VFAs) which can contribute up to 70% of the animal’s energy requirements [23]. During ruminant fermentation of feed materials, CH_4_ is also produced as an end-product by methanogenic archaea or methanogens. As depicted in Fig. 1, these microorganisms have the capacity to convert H_2_ and CO_2_, but also formic acid and methylamines, present in the rumen into CH_4_ [5]. Methanogenesis can be beneficial for the overall rumen as it prevents accumulation of excess H_2_, but it also results in between 2% and 12% loss of energy from feed [6]. Moreover, as described above, the production of enteric CH_4_ by ruminants also contributes significantly to global GHG emissions and thus, in recent years a variety of strategies have been explored to reduce the global burden of CH_4_ produced by ruminants.

Archaea represent 3%–4% of the overall rumen microbiome, where the genus *Methanobrevibacter* is the most dominant, representing approximately 65% of all rumen methanogens [6, 28]. The *Methanobrevibacter* genus comprises two subgroups; the SGMT clade (*Mbb. smithii, Mbb. gottschalkii, Mbb. millerae* and *Mbb. Thaueri)*, and the RO clade (*Mbb. ruminantium* and *Mbb. olleyae).* Overall, the diversity of methanogens in the rumen is low when compared to other microbial populations; by 2017, only 8 species of methanogens had been isolated into pure cultures [6]. There are two main pathways for methanogenesis which differ based on the substrates utilised by methanogens to produce CH_4_. The hydrogenotrophic pathway converts H_2_ and CO_2_ into CH_4_, while the acetoclastic pathway utilises acetate. In both pathways, the rate-limiting step is catalysed by the enzyme methyl-coenzyme M reductase (Mcr). A number of studies have explored the hypothesis that it is the internal composition of the ruminant methanogen community, rather than their overall abundance, that is responsible for CH_4_ production [29]. For example, the SGMT clade of *Methanobrevibacter* contains Mcr isozymes McrI and McrII, which allow the methanogens to utilise greater amount of H_2_. The RO clade, on the other hand, only possesses McrI [30]. Other studies reported that a decrease in proportion of *Methanobrevibacter* populations was associated with decreased CH_4_ emissions [31].

The internal dynamics of methanogens with other microorganisms in the rumen (bacteria, protozoa) also play a part in CH_4_ emissions. The abundance and proportions of ruminant bacteria have been linked with changes in CH_4_ emissions. This is primarily due to certain bacterial species producing more or less H_2_, which is utilised in the hydrogenotrophic pathway in methanogenesis [32]. Hristov et al. [33] reported a 64-fold increase in H_2_ emissions from dairy cows treated with the anti-methanogenic compound 3-NOP, indicating the importance of H_2_ on methanogenesis and thus, how this may be altered by bacteria which produce H_2_. Protozoa in the rumen have also been connected with methanogenesis. Newbold et al. [34] determined that defaunation, the process of removing protozoa from the rumen, decreased CH_4_ emissions by 11%. This decrease in CH_4_ may also be associated with H_2_ production from rumen protozoal hydrogenosomes [35]. However, rumen protozoa also differ markedly in their internal composition between animal microbiomes and thus defaunation may not be a straightforward strategy for CH_4_ reduction. Careful consideration must also be given to overall rumen fermentation efficiency when CH_4_-reduction strategies are applied, including adequate VFA generation, a source of energy for the animal. Moreover, as the generation of CH_4_ requires H_2_, an excess of H_2_ may accumulate in the rumen when the methanogenesis is inhibited [7, 36].

## Exploration of seaweeds as anti-methanogenic agents

To date, most studies have focused on the incorporation of whole seaweed biomass as an anti-methanogenic agent through in vitro studies. This is achieved primarily through batch fermentation or through rumen simulation technique (RUSITEC) [37]. A summary of representative studies examining CH_4_ mitigation through addition of seaweed biomass in vitro during the period 2013–2022 are summarised in Table 1.

**Table 1:** In vitro studies incorporating whole macroalgal biomass in ruminant feed and effects on CH_4_ emission

Seaweed species	Animal	In vitro system	Basal diet	Inclusion rate	Impact on CH_4_ emissions	References
Red seaweeds						
* Asparagopsis taxiformis*	Holstein cows	Ankom gas production system	70% alfalfa 15% dried distillers grain 15% rolled corn	5% dry matter	Reduction of 74% CH_4_ in comparison with control	[38]
* Asparagopsis taxiformis*	Lactating Swedish Red cows	Gas production recorder	Timothy grass, rolled barley, rapeseed in ratio 545:363:92 g/kg diet dry matter	20 g/kg organic matter 0 g/kg 0.06 g/kg 0.13 g/kg 0.25 g/kg 0.5 g/kg 1.0 g/kg diet organic matter	99% CH_4_ inhibition compared to control, when included at 20 g/kg organic matter Dose dependent response observed with CH_4_ emission decreasing curvilinearly	[39]
* Mastocarpus stellatus* * Palmaria palmata* * Porphyra* sp.	Murciano-Granadina goats	Batch fermentation	1:1 oat hay and concentrate (containing cereals at 633 g/kg fresh matter)	84 g/kg fresh matter 130 g/kg fresh matter 150 g/kg fresh matter	No statistically significant reduction in CH_4_ emission observed	[19]
* Halymenia floresii* * Hypnea pannosa*	Brahman steers cattle	Ankom gas production system	Rhodes grass	20% organic matter	No statistically significant reduction in CH_4_ emission observed	[18]
* Asparagopsis taxiformis*	Brahman steers cattle	Ankom gas production system	Rhodes grass	0.5%–10% organic matter	Dose dependent response observed with total inhibition of CH_4_ at dose rates ≥ 2%	[40]
* Bonnemaisonia hamifera* * Euptilota formisissima* * Plocamium cirrhosum* * Vidalia colensoi*	Non-lactating Friesian × Jersey dairy cows	Batch fermentation	Ryegrass hay	0 2% 6% 10% feed organic matter	*Bonnemaisonia hamifera* reduced CH_4_ emission by 17.1% at 2% inclusion rate, 95.4% at 6% inclusion rate, and 98% and 10% of inclusion rate *Euptilota formisissima* and *Plocamium cirrhosum* when included at 10% reduced CH_4_ by 50.5% and 39.5% respectively	[41]
* Chondrus crispus* * Furcellaria* spp.	Lactating Holstein cows	Continuous fermentation vessel	Total mixed ration containing timothy grass, alfalfa, cereals supplemented with vitamins and minerals	0.14 g/d	13% reduction in CH_4_ compared to control 12% reduction in CH_4_ compared to control	[42]
* Asparagopsis taxiformis* * Halymenia floresii* * Hypnea pannosa* * Laurencia filiformis*	Brahman steers cattle	Ankom gas production system	Flinders grass	20% w/w total feed	98.9% reduction of CH_4_ 26.6% reduction of CH_4_ 42.5% reduction of CH_4_ 42.5% reduction of CH_4_	[17]
* Asparagopsis taxiformis*	Brahman steers cattle	Ankom gas production system	Rhodes grass	0–16.7% organic matter	99% reduction of CH_4_ at doses ≥ 2%	[43]
* Gigartina* sp. * Gracilaria vermiculophylla*	Non-lactating Holstein cows	Batch fermentation	Incubated with meadow hay and corn silage	25% dry matter	44% CH_4_ reduction when incubated with meadow hay but not with corn silage 59% CH_4_ reduction when incubated with meadow hay and 63% with corn silage	[14]
* Gracilaria vermiculophylla*	Non-lactating Holstein cows	RUSITEC	Total mixed ration containing 230 g/kg corn silage, 430 g/kg haylage, 150 g/kg wheat straw, 190 g/kg concentrate	25% dry matter	No effect on CH_4_ emission	[44]
* Asparagopsis taxiformis*	One Jersey and one Holstein	RUSITEC	Super basic ration containing 70% alfalfa pellets, 15% rolled corn, 15% dried distillers grains	5% w/w	95% reduction in CH_4_ formation	[45]
* Asparagopsis taxiformis*	Lactating Holstein cows	Ankom gas production system	Total mixed ration	1% dry matter	98% reduction in CH_4_ yield	[16]
* Asparagopsis taxiformis*	Brahman steers cattle	Ankom gas production system	Rhodes grass	2% of organic matter	Post-harvesting processes including rinsing, freezing, drying tested; the frozen then freeze-dried treatment totally inhibited CH_4_ emission	[46]
* Asparagopsis taxiformis*	Brahman steers cattle	Batch fermentation	Rhodes grass	2% of organic matter	> 99% reduction in CH_4_ compared to basal substrate only control	[13]
* Palmaria palmata*	Swedish Red cows	Batch fermentation	Total mixed ration (grass silage/concentrate ratio 600/400 g/kg dry matter basis)	Silage replaced by: 0 g/g 0.15 g/g 0.3 g/g 0.45 g/g dry matter	No effect on CH_4_ emission	[47]
Brown seaweeds						
* Ascophyllum nodosum* * Sargassum fulvellum* * Ecklonia maxima* * Lessonia flavicans* * Lessonia nigrescens* * Laminaria japonica*	Non-lactating cows	Batch fermentation	50:50 grass hay:concentrates	Two inclusion rates tested: As a feed additive each seaweed was added in addition to basal diet, at 20%. As feed where each seaweed replaced 20% of concentrates in basal diet	As feed additive no effect on CH_4_ was observed As feed CH_4_ was reduced by 18% in the case of *E. maxima* and 21% in the case of *L. japonica*	[48]
* Ascophyllum nodosum* * Laminaria digitata*	Holstein-Friesian cows	RUSITEC	50:50 forage:concentrates	5% dry matter	No effects on CH_4_ emissions	[49]
* Zonaria farlowii*	Holstein cow	Ankom gas production system	70% alfalfa 15% dried distillers grain 15% rolled corn	5% dry matter	11% reduction in CH_4_	[38]
* Undaria pinnatifida* * Sargassum fusiforme* * Sargassum fulvellum*	Non-lactating Hanwoo cows	Batch fermentation	300 mg timothy hay 200 mg corn grain	0.25 mg/mL	*Undaria pinnatifida* reduced CH_4_ emission by 26.8% at 12 h and 21.3% at 24 h *Sargassum fusiforme* reduced CH_4_ emission by 23.4% at 12 h and 24.4% at 24 h *Sargassum fulvellum* reduced CH_4_ emission by 26.3% at 12 h and 24.6% at 24 h	[50]
* Ecklonia stolonifera* * Eisenia bicyclis* * Sargassum fulvellum* * Undaria pinnatifida* * Sargassum fusiforme*	Holstein cows	Batch fermentation	Timothy grass	5% dry matter	For all seaweeds except *Sargassum fusiforme* CH_4_ emission increased at 6 and 24 h incubation After 48 h, CH_4_ reduced by 36.1% for *Ecklonia stolonifera*, 32.4% for *Eisenia bicyclis*, 10.4% for *Sargassum fulvellum*, 26.7% for *Undaria pinnatifida*, and 13.9% for *Sargassum fusiforme*	[51]
* Cystoseira trinodis* * Padina australis* * Dictyota* spp.	Brahman steers cattle	Ankom gas production system	Rhodes grass	20% feed total (diet organic matter)	*Cystoseira trinodis* significantly reduced CH_4_ emission	[18]
* Cystoseira trinodis* * Dictyota bartayresii* * Padina australis* * Sargassum flavicans*	Brahman steers cattle	Ankom gas production system	Rhodes grass	0.5%–10% organic matter	No significant reduction in CH_4_ observed	[40]
* Cystoseira trinodis* * Dictyota bartayresii* * Hormophysa triquetra* * Padina australis* * Sargassum flavicans* * Colpomenia sinuosa*	Brahman steers cattle	Ankom gas production system	Flinders grass	20% w/w total feed	Strongest CH_4_ inhibition observed from *Dictyota bartayresii*, inhibiting CH_4_ by 92.2%	[17]
* Laminaria ochroleuca* * Saccharina latissima*	Non-lactating Holstein cows	Batch fermentation	Incubated with meadow hay and corn silage	25% dry matter	With meadow hay, no change in CH_4_ emission With corn silage, addition of *Laminaria ochroleuca* increased CH_4_ emission	[14]
* Saccharina latissima*	Non-lactating Holstein cows	RUSITEC	Total mixed ration containing 230 g/kg corn silage, 430 g/kg haylage, 150 g/kg wheat straw, 190 g/kg concentrate	25% dry matter	No effect on CH_4_ emission	[44]
* Sargassum horneri*	Holstein cows	Batch fermentation	Total mixed ration containing grain, cereal meal, alfalfa hay	0 0.5% 1% 2% 3% 4% dry matter	CH_4_ emission was decreased at 24 h after inclusion of *S. horneri*	[52]
* Saccharina latissima* * Alaria esculenta*	Swedish Red cows	Batch fermentation	Total mixed ration (grass silage/concentrate ratio 600/400 g/kg dry matter basis)	Silage replaced by: 0 0.15 0.3 0.45 g/g dry matter	Reduction in CH_4_ emission with increasing inclusion of *A. esculenta*	[47]
Green seaweeds						
* Caulerpa lentillifera* * Caulerpa taxifolia* * Cladophora coelothrix* * Ulva ohnoi* * Cladophora patentiramea* * Ulva* sp. * Derbesia tenuissima*	Brahman steers cattle	Ankom gas production system	Rhodes grass	20% feed total organic matter	No significant change in CH_4_ emission observed	[18]
* Caulerpa taxifolia* * Cladophora patentiramea* * Ulva ohnoi*	Brahman steers cattle	Ankom gas production system	Rhodes grass	0.5%–10% organic matter	No significant change in CH_4_ emission observed	[40]
* Caulerpa taxifolia* * Chaetomorpha linum* * Cladophora coelothrix* * Cladophora patentiramea* * Derbesia tenuissima* * Ulva sp.* * Ulva ohnoi*	Brahman steers cattle	Ankom gas production system	Flinders grass	20% w/w total feed	Addition of *Derbesia tenuissima* increased CH_4_ emission Addition of *Cladophora patentiramea* reduced CH_4_ by 66.3% compared to control	[17]
* Ulva sp*	Non-lactating Holstein cows	Batch fermentation	Incubated with meadow hay and corn silage	25% dry matter	When incubated with meadow hay CH_4_ decreased by 55% When incubated with corn silage no reduction observed	[14]
* Ulva rigida*	Non-lactating Holstein cows	RUSITEC	Total mixed ration containing 230 g/kg corn silage, 430 g/kg haylage, 150 g/kg wheat straw, 190 g/kg concentrate	25% dry matter	No effect on CH_4_ emission	[44]
* Ulva species B* (taxonomically unresolved cultivated species)	Non-lactating Friesian × Jersey dairy cows	Batch fermentation	Ryegrass hay	0 2% 6% 10% organic matter	No effect on CH_4_ emission	[41]
* Ulva* sp.	Holstein cows	Batch fermentation	Total mixed ration containing grain, cereal meal, alfalfa hay	0 0.5% 1% 2% 3% 4% dry matter	4% inclusion of *Ulva* sp. reduced CH_4_ significantly in comparison with control	[52]

### Red seaweeds

**Fig. 2:**
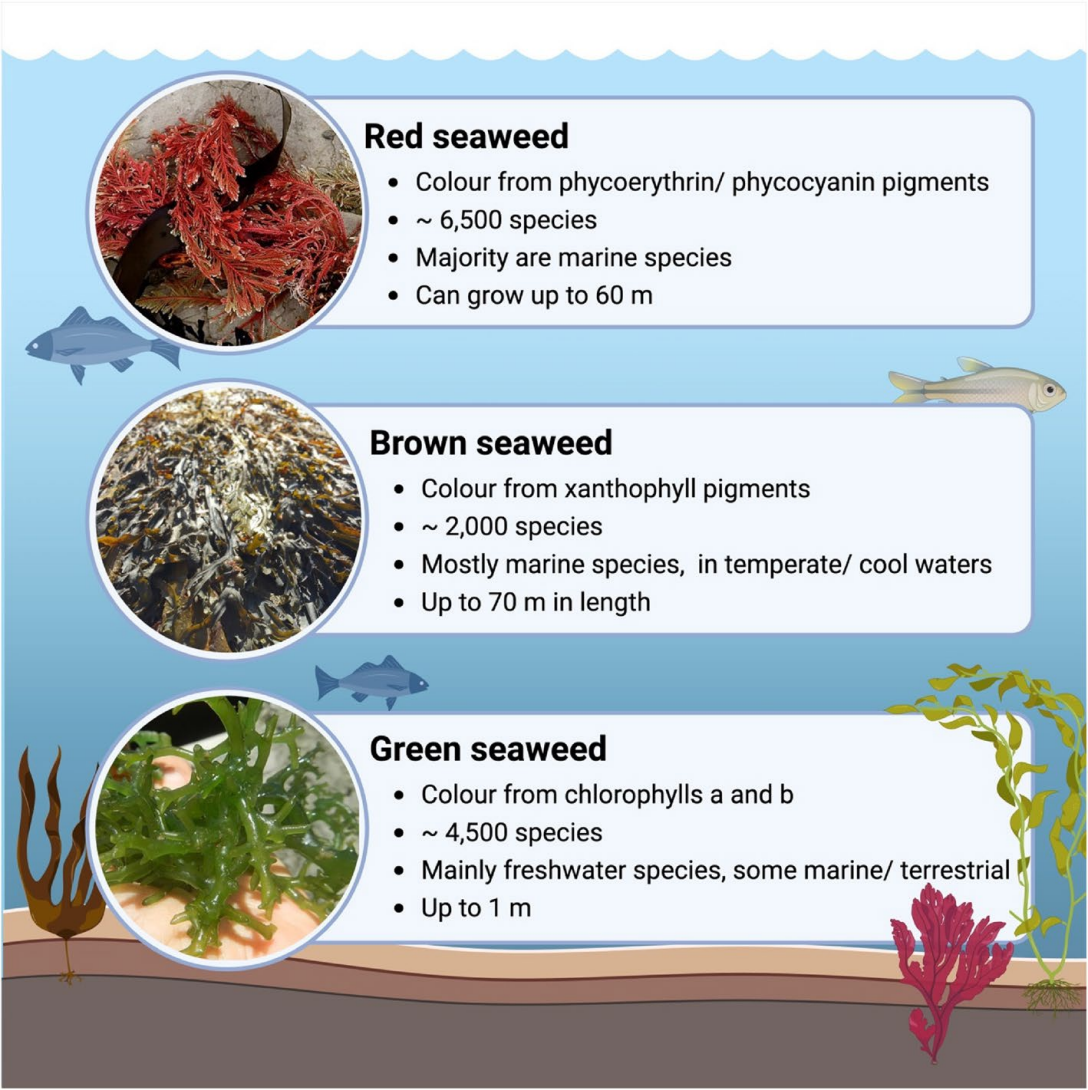
Summary of main biological characteristics of the 3 main groups of seaweeds: Red macroalgae (Rhodophyta), brown macroalgae (Phaeophyceae), and green macroalgae (Chlorophyta). Information was summarised from [53–55]. Image created on BioRender.com

Red macroalgae (Rhodophyta) comprise approximately 6,500 species (see Fig. 2) most commonly found in intertidal zones in marine environments [56], and are an abundant source of a variety of bioactive compounds including carotenoids, phycobiliproteins and polysaccharides [53]. A wide variety of biological activities have been reported from red macroalgae including antimicrobial, anti-inflammatory, antioxidant, and anti-tumour, most frequently linked to the oligosaccharides produced by these species [57]. The gelling properties of these compounds, mainly carrageenan and agar, have contributed to the expansion of the use of these seaweeds by the food processing industries [58]. Moreover, red seaweeds are also important producers of halogenated compounds, such as bromoform, which has been investigated for its anti-methanogenic activity [13, 26]. The majority of studies exploring the anti-methanogenic effects of red seaweeds focus on the species *A. taxiformis* (native to Asia and Australia), but also *Palmaria palmata* and *Gracilaria vermiculophylla* [14, 47].

*A. taxiformis* has emerged as a seaweed with particular potential to reduce ruminant CH_4_ emissions; multiple studies compiled in Table 1 reported a reduction in CH_4_ emission of > 95% when using this species during in vitro studies [13, 16, 17, 39, 45]. Chagas et al. [39] reported 99% CH_4_ inhibition compared to the control, when *A. taxiformis* was included at 20 g/kg organic matter in an in vitro study using rumen fluid from lactating Swedish Red cows. Kinley et al. [40] applied *A. taxiformis* in a dose-dependent manner from 0.5% to 10% organic matter using an in vitro gas recorder system with rumen inoculum from Brahman steers cattle. At concentrations equal to or above 2%, complete inhibition of CH_4_ was reported. Stefenoni et al. [16] incorporated *A. taxiformis* at 1% dry matter to a basal feed of total mixed ration and analysed effects on rumen fermentation using an in vitro gas production system with rumen inoculum from lactating Holstein cows. This concentrated of *A. taxiformis* yielded a 98% reduction in CH_4_ compared to the control, measured using gas chromatography. The ability of this *A. taxiformis* to reduce CH_4_ emissions has been linked to the secondary metabolite bromoform which is produced in large amounts by this seaweed species [7, 27]. *G. vermiculophylla* is another red seaweed which has been studied for its potential to reduce CH_4_ emissions, with mixed results. Maia et al. [14] found that the incorporation of this seaweed as a feed additive (at 25% dry matter) reduced CH_4_ emissions by 59% compared to the control with meadow hay as a basal feed, and 63% when corn silage was used. However, the same group in 2019 found no effect on CH_4_ emissions when including *G. vermiculophylla* at 25% dry matter rate and a basal feed of total mixed ration containing corn silage [44]. Certain studies have shown a dose dependent response of seaweed addition to feed [39, 40], with Mihaila et al. [41] reporting a 17% reduction in CH_4_ with the addition of 2% *Bonnemaisonia hamifera*, and a 95% CH_4_ reduction when the same species was included at 6% feed. This study determined that *B. hamifera* did not contain bromoform, despite having a high level of bromine. This may indicate that other brominated compounds besides bromoform may have strong anti-methanogenic capabilities.

Multiple studies compare different seaweed species for their potential to reduce CH_4_ in vitro [19, 38, 42]. Machado et al. [17] explored 20 species of marine and freshwater algae (listed in Table 1) for their potential in vitro CH_4_ reduction activity, incorporating seaweed at 20% w/w Flinders grass basal feed and rumen inoculum from Brahman steers, using an in vitro gas production system. The authors found that, in general, marine algae were more effective in reducing CH_4_ than freshwater algae, with *A. taxiformis* reducing CH_4_ emissions by 98% in comparison with the control. Less pronounced CH_4_ reductions were observed from the red macroalgae *Halymenia floresii* (26% CH_4_ reductions in comparison with control), *Hypnea pannosa* (42%), and *Laurencia filiformis* (39%). de la Moneda et al. [19] also compared a variety of seaweeds, including the red seaweeds *Mastocarpus stellatus, P. palmata, Porphyra *sp. harvested at spring and autumn, for their potential to reduce CH_4_ in vitro using rumen inoculum from Murciano-Granadina goats. The seaweeds were included at a range of concentrations (84 g/kg, 130 g/kg and 150 g/kg fresh matter) to a basal feed of 1:1 oat hay and concentrate (containing cereals at 633 g/kg fresh matter). In all cases, no statistically significant reduction in CH_4_ was reported.

As well as comparing between different seaweed species, the effects of different seaweed processing techniques have been explored for effects on CH_4_ reduction in vitro. While most studies use seaweed that has been freeze-dried, Vucko et al. [46] prepared the red seaweed *A. taxiformis* at 2% dietary inclusion rate using a variety of post-harvesting methods. This study used combinations of post-harvest techniques (namely rinsing, freezing, and drying) in a factorial design on the seaweed biomass and examined the effects of CH_4_ emission. All treatments which were frozen and then freeze dried, regardless of rinsing, completely inhibited CH_4_ emission. Of the treatments which inhibited CH_4_ emissions, the group which was frozen and freeze dried (without rinsing) contained the highest amount of bromoform (4.39 ± 0.07 mg/g dry weight (DW)), which is often used as an indicator of overall CH_4_ reduction capabilities.

Overall, mixed results are observed from the major relevant studies on in vitro use of red seaweed as a feed additive to reduce CH_4_ emissions. As detailed in Table 1, certain studies (particularly those using *A. taxiformis)* have shown near complete inhibition of CH_4_ [16, 17, 39, 43]. Brooke et al. [38] observed less CH_4_ inhibition, with a reduction of 74% CH_4_ in comparison with the control in treatments containing *A. taxiformis*, compared to other studies reporting > 95% inhibition. Other studies report moderate CH_4_ reduction; *B. hamifera* reduced CH_4_ by 17% [41], *Chondrus crispus* and *Furcellaria* spp. reduced CH_4_ emission by 13% and 12% respectively [42], and Machado et al. [17] reported reductions of 26%–42% CH_4_ with the addition of a variety of red seaweeds (*H. floresii, H. pannosa, L. filiformis*). However, multiple studies report no effect on CH_4_ emissions [18, 19, 44, 47]. Further research is needed to determine whether these effects are due to the particular seaweed species and associated bioactive compounds contained within the biomass, or whether it is due to other factors including dosage rate, post-harvesting treatment of the biomass, or bioavailability of the relevant secondary metabolites.

Within the studies reporting complete or near complete inhibition of CH_4_ with the addition of *A. taxiformis*, the dosage of biomass used should also be standardised. Kinley et al. [40], Machado et al. [43] determined that *A. taxiformis* must be included at a minimum inclusion rate of 2% to basal feed to totally inhibit CH_4_. The same group quantified the amount of bromoform in *A. taxiformis* when it was added to Rhodes grass basal feed. They found that the minimum amount of bromoform necessary to totally inhibit CH_4_ emission was 1 mg/g DW of bromoform in 2% *A. taxiformis* [46]. This method of bromoform quantification may be used to standardise the dosage of *A. taxiformis* treatment as a feed additive. Standardisation such as this is vital if the addition of macroalgae as a feed additive is to be successful at scale, particularly if seaweeds may differ in their amount of bromoform.

### Brown seaweeds

While the majority of CH_4_ reduction studies in vitro are focused on the red seaweed *A. taxiformis*, brown seaweeds (Phaeophyta) are becoming increasingly studied for this purpose. Found in temperate marine environments (see Fig. 2) [5], brown seaweeds are historically the most consumed type as food products globally [59]. Brown seaweeds are known to possess a wide variety of bioactive compounds including polysaccharides (can comprise up to 70% DW), such as fucoidan and laminarin, which have been extensively studied for their nutraceutical and therapeutic properties [54]. Brown seaweeds are the only type of seaweeds to produce phlorotannins, a heterogenous group of molecules which can constitute up to 90% of the phenolic composition of brown seaweeds [60] with reported antimicrobial, antioxidant, and anti-inflammatory properties [61–63].

Wang et al. [64] reported a significant reduction in CH_4_ emissions (*P* < 0.001) from a phlorotannin-rich extract of the brown seaweed *A. nodosum* using in vitro batch fermentation, where the extract of *A. nodosum* was added to achieve a concentration of 500 µg phlorotannin/mL of medium containing a basal feed of barley silage and alfalfa hay. Belanche et al. [49] however, reported no effect on CH_4_ emission when utilising either *A. nodosum* or *Laminaria digitata* at 5% dry matter in a RUSITEC apparatus with rumen inoculum from Holstein-Friesian cows. Other species of brown seaweed that have been explored for potential anti-methanogenic activity include *Saccharina, Sargassum*, *Ecklonia* and *Cystoseira* spp. [18, 44, 50, 51]. Machado et al. [17] reported CH_4_ inhibition that is comparable to the largest reductions seen from *A. taxiformis*, with *Dictyota bartayresii* inhibiting CH_4_ by 92% when applied at 20% w/w total feed. Moderate CH_4_ inhibition was reported by Choi et al. [51] utilising brown seaweeds. *Undaria pinnatifida* reduced CH_4_ emission by 26% at 12 h and 21% after 24 h, *Sargassum fusiforme* reduced CH_4_ emission by 23% at 12 h and 24% at 24 h, and *Sargassum fulvellum* reduced CH_4_ emissions by 26% at 12 h and 24% at 24 h. As with red seaweeds, a dose-dependent response has been reported with brown seaweeds, with higher concentrations of *Alaria esculenta* (tested at 0, 0.15, 0.3, and 0.45 g/g dry matter in vitro) resulting in greater reductions in CH_4_ [47]. However, other studies report no reduction of CH_4_ emissions after the incorporation of brown seaweed as a feed additive [14, 44, 65]. Dubois et al. [18] found *Cystoseira trinodis* reduced CH_4_ emissions when incorporated at 20% organic matter, while Kinley et al. [65] and Machado et al. [17] found no impact of *C. trinodis* on CH_4_ emission, even when applied at the same dosage rate in vitro. Further research into these particular macroalgae species and in vitro studies are needed to ensure reproducibility of studies, and adoption of macroalgae as an anti-methanogenic agent at a global scale.

Ahmed et al. [48] explored the dosage rate of brown macroalgae and its effects on CH_4_ emissions. In this study a variety of brown seaweeds (*A. nodosum, S. fulvellum, Ecklonia maxima, Lessonia flavicans, Lessonia nigrescens, and Laminaria japonica*) were analysed for their effect on ruminal fermentation and CH_4_ emissions in vitro with rumen inoculum from non-lactating cows. Seaweeds were incorporated either as a feed (where the seaweed replaced 20% of the basal diet of 50:50 grass hay:concentrates) or a feed additive (where the seaweed was added in addition to the basal diet, at a dosage rate of 20% of the basal diet). The authors reported that when the seaweed was used as a feed additive, no effects on CH_4_ were observed. However, when the seaweed replaced 20% of the basal feed, a reduction in CH_4_ was observed when using several seaweeds; CH_4_ reduction of 18% in comparison with the control was observed when using *E. maxima* and reduced by 21% when using *L. japonica.* Further research should potentially explore the different permutations of seaweeds and basal feed concentrations, as these may inform the overall potential of incorporating seaweeds to reduce ruminant CH_4_ emissions. The authors also reported a reduction in overall rumen fermentation and a decrease in production of VFA, in the treatments where seaweed was applied as feed [48]. Therefore, it is unclear whether the reduction in CH_4_ is due to an anti-methanogenic bioactive compound within the brown seaweeds, or simply a result of an overall decrease in rumen fermentation efficiency.

In all studies, it is crucial to monitor fermentation kinetics, overall digestibility and fermentation profile in the rumen when considering incorporating seaweeds as a feed or feed additive. Phlorotannins from brown seaweeds have been shown to negatively affect ruminal fermentation [66] and may have particular effects against *Fibrobacter succinogenes* which degrades fibre [48]. Further research is required in order to identify what may be a variety of outcomes (particularly those affecting the microbial communities) in the rumen when seaweeds are incorporated as ruminant feed/feed additive. Further in vitro and in vivo studies should be carried out to ascertain the optimal dosage concentrations and techniques to ensure rumen fermentation efficiency is retained while using alternative feeds. Park et al. [52] reported a decrease in ruminant CH_4_ in vitro (using rumen inoculum from Holstein cows) when incorporating the brown seaweed *Sargassum horneri* at 4% dry matter to basal feed of total mixed ration containing grain, cereal meal and alfalfa hay, with no adverse effects on VFA production. Therefore, the addition of *S. horneri* may have a targeted effect on rumen methanogens and may be a suitable candidate for incorporation into ruminant feed to reduce CH_4_ emissions. The authors hypothesise that this effect may be due to phlorotannins in *S. horneri*, but further experiments are required to investigate this.

### Green seaweeds

Green macroalgae can be found in fresh water as well as marine environments (see Fig. 2), of which the species *Ulva* is one of the main representatives, and is often reported in ‘green tides’ eutrophication in coastal regions [67]. Green seaweeds are reported to have relatively high protein (10%–30%) and polysaccharide contents (15%–65% in certain *Ulva* spp.) [55]. Certain polysaccharides found in green seaweeds have been studied for biological activities including the sulfated polysaccharide ulvan [68] which has been found to possess antiviral, antioxidant, anticancer properties [69] as well as being utilised in biomaterials and as a feedstock for biofuel production [70].

The main species of green seaweeds that have been explored for their anti-methanogenic potential include *Ulva*, *Cladophora* and *Caulerpa* spp. Park et al. [52] examined the effects in vitro (using rumen inoculum from Holstein cows) of including *Ulva* sp. in ruminant feed. The authors tested a variety of inclusion rates ranging from 0.5% to 4% dry matter added to basal feed of total mixed ration containing grain, cereal meal, alfalfa hay, and found that *Ulva* sp. included to basal feed at 4% dry matter reduced CH_4_ compared to the control. However, total VFAs were reduced with the addition of seaweed, therefore further study is required to ascertain if this species is a suitable candidate for CH_4_ reduction. Maia et al. [14] analysed 5 species of red, brown, and green seaweeds, including *Ulva* sp., using in vitro batch fermentation with non-lactating Holstein cows ruminal fluid. *Ulva* sp. incorporated at 25% dry matter yielded a 55% decrease in CH_4_ in comparison with the control when using this seaweed with a basal diet of meadow hay. However, when *Ulva* sp. was included in a basal diet of corn silage, no effect on CH_4_ was appreciated observed by the authors. This indicates the impact the basal diet can have on the overall effects of incorporating seaweed into ruminant feed. Machado et al. [43] reported the same phenomenon, whereby a reduction in CH_4_ emissions was reported when seaweed was included in basal diets that were high in protein (as opposed to diets which were not protein-rich). As has been observed with other in vitro studies, mixed results of CH_4_ reduction are reported using green seaweeds. Maia et al. [44] reported no effect on CH_4_ emissions after the inclusion of *Ulva rigida* at 25% dry matter in ruminant feed during an in vitro trial using RUSITEC apparatus with ruminal fluid of non-lactating Holstein cows. Mihaila et al. [41] also observed no effect on CH_4_ emissions after including *Ulva* sp. to ryegrass hay basal feed at a variety of concentrations (2%–10% organic matter) using in vitro batch fermentation. Machado et al. [17] did observe a 66% reduction in CH_4_ in comparison with the control after in vitro inclusion of *Cladophora patentiramea* at 20% w/w total basal feed (flinders grass). This study analysed 20 species of marine and freshwater algae (listed in Table 1), and of the seven green seaweed species tested (*Caulerpa taxifolia*, *Chaetomorpha linum*, *Cladophora coelothrix*, *C. patentiramea*, *Derbesia tenuissima*, *Ulva *sp.*, Ulva ohnoi*), the treatment with *C. patentiramea* resulted in the lowest CH_4_ emissions. The treatment with *D. tenuissima* had the least effect on CH_4_ emissions, with CH_4_ emissions from this treatment nearly as much as the control. Kinley et al. [65] and Dubois et al. [18] also carried out in vitro screening of a number of seaweed species and their potential to reduce CH_4_ (*Caulerpa lentillifera, C. taxifolia, C. coelothrix, U. ohnoi*, *C. patentiramea*, *Ulva* sp., *D. tenuissima* and *C. taxifolia, C. patentiramea*, *U. ohnoi* respectively) using rumen inoculum from Brahman steers cattle, with seaweed incorporated to Rhodes grass basal feed at 20% organic matter. Of these species of green seaweed studied, no changes in CH_4_ emissions were observed by any seaweed. Overall, studies using red or brown seaweeds report stronger reductions in CH_4_ emissions than studies using green seaweeds.

## Bioactive compounds with anti-methanogenic activities from seaweeds

The main bioactive compounds identified as contributing to the anti-methanogenic activities of seaweeds are bromoform and other halogenated compounds mainly from red seaweeds; phenolic compounds, such as phlorotannins from brown seaweeds, and saponins. *A. taxiformis*, the most frequently studied species with anti-methanogenic properties, contains a variety of halogenated compounds, such as bromoform, usually the most abundant anti-methanogenic secondary metabolite; but also dibromochloromethane, bromochloroacetic acid, and dibromoacetic acid [71]. These halogenated compounds reduce CH_4_ emission through interference with the biochemical pathways involved in methanogenesis [10]. Other secondary metabolites from seaweeds, such as phlorotannins and saponins have been shown to reduce CH_4_ emissions [64, 72] through direct interactions with archaea and through reduction of rumen protozoa which are linked to methanogenesis, but further study is necessary to elucidate the mechanisms of action and overall effects of these metabolites on rumen fermentation efficiency.

### Bromoform and halogenated compounds

**Fig. 3:**
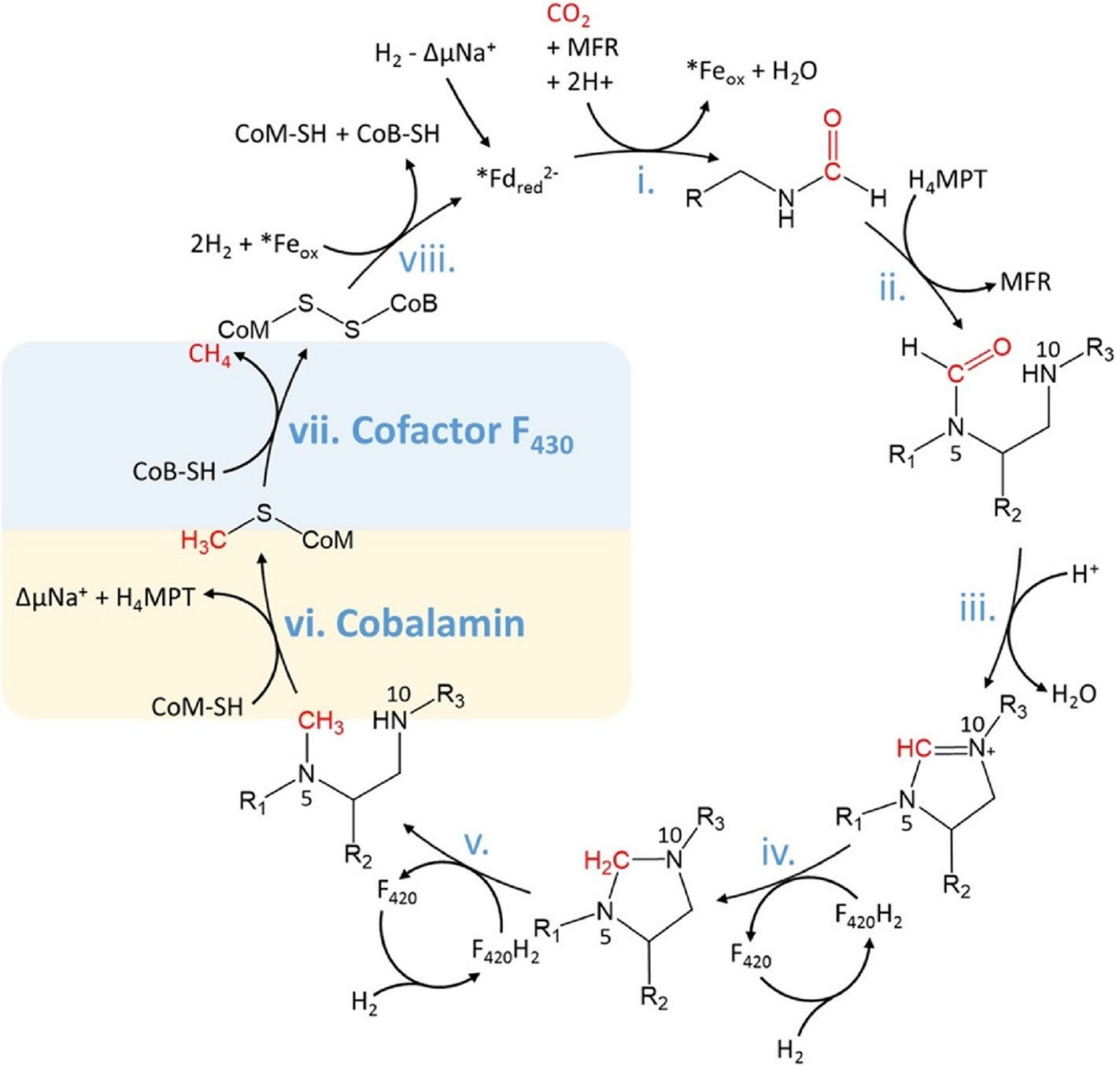
Methanogenesis via hydrogenotrophic pathway in methanogens (the Wolfe cycle), depicted via steps (i) to (viii). (i): Reaction of CO_2_ with methanofuran (MFR) to produce formyl-MFR. (ii): Formyl group moves to tetraydromethanopterin (H_4_MPT). (iii), (iv), (v): Formation of imine and reduction reactions. (vi): Methyl transfer from methyl-H4MPT to CoM-SH (reaction catalysed by coenzyme M (CoM) methyl-transferase). (vii): Methyl group (CH_3_) reduced to methane (reaction catalysed by methyl-CoM reductase (cofactor F_430_)). (viii): CoM regenerated via ferredoxin. Image reproduced with permission from [7]

A number of studies report the reduction of ruminant CH_4_ by halogenated compounds present in red seaweeds, most commonly bromoform [13, 27], as summarised in Table 2. These compounds inhibit methanogenesis in the rumen via disruption of enzymes in this biochemical pathway, namely competitive inhibition of cobamide-dependent coenzyme M methyltransferase (step (vi) in Fig. 3) which inhibits methyl transfer, and blocking Mcr (step (vii) in Fig. 3), the enzyme that catalyses the rate-limiting step in methanogenesis [7, 73, 74]. Quantification of bromoform/halogenated compounds in seaweeds has been used as a proxy to estimate the reduction in CH_4_ that may be expected during treatment with these seaweeds, and this proxy could be implemented to standardise the inclusion levels of seaweed in agricultural settings. Min et al. [9] presented a polynomial correlation between bromoform concentration and CH_4_ emissions (in vitro), where it was shown that at bromoform concentrations above ~ 0.25 mg/g organic matter, CH_4_ decreases linearly with increasing bromoform concentration until ~ 0.8 mg/g bromoform, where CH_4_ emission reaches zero.

**Table 2:** Studies analysing effects of bromoform/halogenated compounds on CH_4_ emissions in vitro and in vivo

Compound	Type of study	Animal	Basal diet	Dosage	Impact on CH_4_ emissions	References
Bromoform	In vitro (batch fermentation)	Brahman steers cattle	Rhodes grass	2 concentrations tested: 1 µmol/L and 5 µmol/L	Addition of 1 µmol/L reduced CH_4_ by 77% and 5 µmol/L reduced CH_4_ by > 99% compared to basal substrate-only control	[13]
Bromoform	In vitro (gas production recorder)	Lactating Swedish Red cows	Timothy grass, rolled barley, rapeseed in ratio 545:363:92 g/kg diet dry matter	2 dosage rates: 1.5 mg/g dry matter, and 3 mg/g dry matter	95% reduction in CH_4_	[39]
Bromoform Dibromochloromethane Bromochloroacetic acid Dibromoacetic acid	In vitro (Ankom system)	Brahman steers cattle	Rhodes grass hay	4 concentrations tested: 1 µmol/L, 5 µmol/L, 10 µmol/L, and 25 µmol/L	In the case of bromoform and dibromochloromethane, CH_4_ production completely inhibited at concentrations ≥ 5 µmol/L In the case of bromochloroacetic acid and dibromoacetic acid, no effect on CH_4_ production observed	[71]
Bromochloromethane	In vivo	Murciano-Granadina lactating goats	Alfalfa hay with 600 g/d concentrates	0.3 g of BCM/100 kg body weight BCM administered 2 equal doses per day from parturition to 2 weeks postweaning	33% reduction in CH_4_	[29]
Bromochloromethane	In vivo	Brahman-crossbred steers	Rhodes grass and 1 kg/d proprietary grain pellets	0.3 g of BCM/100 kg body weight	30% reduction in CH_4_	[31]
Bromochloromethane	In vitro (batch and continuous fermentation)	Non-lactating Friesian-Holstein cattle	Batch: hay Continuous: 20 g/d hay	Batch: 5 µmol/L and 10 µmol/L tested Continuous: 5 µmol/L	Batch: 89%–94% reduction of CH_4_ at both concentrations tested Continuous fermentation resulted in 85%–90% CH_4_ reduction	[75]
Bromochloromethane	In vivo	Japanese native (Shiba) goats	50% timothy gras 50% concentrates	Animals sequentially adapted to low, medium, and high doses of BCM. Low dose: 0.5 g/100 kg live weight Medium dose: 2 g/100 kg live weight High dose: 5 g/100 kg live weight	Low dose: 5% reduction in CH_4_ Medium dose: 71% reduction in CH_4_ High dose: 91% reduction in CH_4_	[76]

Machado et al. [13] reported that bromoform applied at a concentration of 5 µmol/L to a basal diet of Rhodes grass in vitro reduced CH_4_ by > 99% compared to basal diet-only control. A dose-dependent response was observed in this study whereby a lesser concentration (1 µmol/L) of bromoform reduced CH_4_ by 77%. The authors also reported that whole *A. taxiformis* biomass included at 2% had the same effect on CH_4_ reduction as bromoform at 5 µmol/L, indicating that whole macroalgal biomass can be used to reduce CH_4_ effectively without the need of further processing to extract bromoform. Interestingly, the bromoform concentration in the *A. taxiformis* biomass used in this study was estimated to be ~ 1.3 µmol/L, yet the whole macroalgal biomass had greater CH_4_-mitigating effect than isolated bromoform applied at a similar concentration (1 µmol/L). Thus, bromoform present within *A. taxiformis* may either be more potent or may act in synergy with other bioactive compounds in this seaweed to provide a greater CH_4_-mitigation effect than the compound on its own. Chagas et al. [39] evaluated a variety of dietary strategies to reduce ruminant CH_4_ emissions in vitro, including bromoform at two inclusion levels; 1.5 mg/g dry matter and 3 mg/g dry matter (added to basal feed of Timothy grass, rolled barley, rapeseed in ratio 545:363:92 g/kg diet dry matter). Bromoform reduced predicted in vivo CH_4_ emissions by 95% in comparison with the control, with *A. taxiformis* biomass reducing predicted in vivo CH_4_ by 99%. These results agree with those reported by Vucko et al. [46], who reported that a minimum threshold of 1 mg/g DM bromoform is necessary for CH_4_ inhibition. Machado et al. [71] identified the bioactive compounds present in a dichloromethane extract of *A. taxiformis* that when used at a dose equivalent to 2% dry matter (added to basal feed of Rhodes grass) reduced CH_4_ by 79% in vitro using rumen inoculum from Brahman steers cattle. The halogenated compounds identified in this extract were bromoform, comprising 1,723 µg/g dry weight of *A. taxiformis* extract, dibromochloromethane (15.8 µg/g DW), bromochloroacetic acid (9.8 µg/g DW), and dibromoacetic acid (15.8 µg/g DW). Each individual compound was then tested in vitro for anti-methanogenic activity, with 4 concentrations tested: 1, 5, 10, and 25 µmol/L added to Rhodes grass and using rumen inoculum from Brahman steers cattle. Bromoform and dichloromethane completely inhibited CH_4_ at concentrations ≥5 µmol/L [71]. While bromoform was the most abundant bioactive compound identified from *A. taxiformis* in this case, the authors also suggested that multiple compounds within *A. taxiformis* could be working synergistically to reduce CH_4_. The authors also noted that rumen fermentation efficiency and VFA production were not hindered with the application of either *A. taxiformis* or bromoform at concentrations > 10 µmol/L.

Other halogenated compounds investigated for anti-methanogenic activity include bromochloromethane (BCM) which has been shown to reduce CH_4_ emissions in vitro and in vivo [75, 77]. The anti-methanogenic activity of BCM is also attributed to inhibiting the methyl transfer step of methanogenesis which is cobamide-dependent [78]. Abecia et al. [29] reported a 33% reduction in CH_4_ emissions in comparison with the control from goats when BCM was included in feed at a dosage rate of 0.3 g BCM/100 kg body weight. The authors did not report any adverse effects on overall rumen fermentation, and actually reported a 36% increase in milk yield, attributed to a shift in fermentation towards propionate rather than acetate. Denman et al. [31] found a similar level of CH_4_ reduction with a similar dosage rate of 0.3 g BCM/100 kg body weight, resulting in 30% CH_4_ reduction in cattle in an in vivo trial. Goel et al. [75] investigated the anti-methanogenic activity of BCM in vitro, comparing batch and continuous fermentation. Batch fermentation resulted in 89%–94% CH_4_ reduction, at 5 and 10 µmol/L BCM. Continuous fermentation was carried out with 5 µmol/L BCM administered once per day for a total of 9 d and resulted in 85%–90% CH_4_ inhibition. Mitsumori et al. [76] explored 3 dosage levels of BCM in an in vivo trial using goats. The animals were sequentially adapted to low (0.5 g/100 kg animal weight), medium (2 g/100 kg) and high (5 g/100 kg) doses of BCM in the diet. A dose-dependent response in CH_4_ reduction was observed by the authors, with the animals receiving a low dose resulting in 5% CH_4_ reduction, the medium dose resulted in 71% CH_4_ reduction and the highest dose of BCM caused 91% CH_4_ inhibition [76]. BCM has been found to be effective at reducing CH_4_ emissions both in vitro and in vivo; however, as it is classed as an ozone-depleting substance its use is controlled in many jurisdictions globally according to the United Nations Montreal Protocol on ozone-depleting substances [79]. Tomkins et al. [77] note that, while the controlled substances such as BCM may be prohibited, studies into the anti-methanogenic efficacy of BCM have served as a proof-of-concept so that similar compounds with comparable mechanisms of action may be useful as anti-methanogenic agents in agricultural settings.

### Tannins and phlorotannins

Tannins from terrestrial plants have previously been shown to reduce ruminant CH_4_ emissions [80–83]. Grainger et al. [81] tested two dosage levels of condensed tannins from the terrestrial plant *Acacia mearnsii* in an in vivo trial using 60 lactating dairy cows; a lower level of tannins (163 g/d) reduced CH_4_ emissions by 14%, while a higher dose (326 g/d) reduced CH_4_ emissions by 29% in comparison with the control. The authors also reported adverse effects of treatment with condensed tannins on milk production, particularly at the higher dosage rate of tannins. Anti-methanogenic activity from tannins has been linked to direct inhibitory effects on methanogens, as well as inhibition of rumen protozoa [84], with *Methanobrevivacter* spp. abundance decreasing with an increased concentration of tannins [82]. Promising results from studies examining the anti-methanogenic potential of terrestrial tannins may encourage further study into macroalgae-derived tannins, such as phlorotannins.

Phlorotannins are polyphenolic compounds consisting of repeating phloroglucinol units which are found only in brown seaweeds [85]. The phlorotannin content of different seaweeds can vary considerably depending on a variety of biotic and abiotic factors including species, location, salinity, UV radiation, age and reproductive status, with reported phlorotannin contents ranging from 0 to 14% dry weight of the seaweed [60]. Wang et al. [64] investigated the effects of phlorotannins from *A. nodosum* on digestion and methanogenesis. An extract of *A. nodosum* was applied in an in vitro batch fermentation with mixed forage and barley grain diets to yield concentrations of 0, 125, 250, or 500 µg phlorotannin/mL in each treatment. CH_4_ emission was reduced over 24 h in treatments supplemented with phlorotannins in comparison with the control; however, the overall fermentation process was adversely affected. Gas production and digestibility were reduced at ≥10 and 100 µg/mL phlorotannins for mixed forage and barley grain diets, respectively. The authors suggested that phlorotannins from *A. nodosum* formed complexes with proteins in the rumen, as previously described in the case of tannins, and that different microbial populations in the rumen may have varying sensitivities to phlorotannins [64]. Certain studies have examined the effects of brown seaweeds and phlorotannins on the ruminant microbiome. Wang et al. [66] added an extract of *A. nodosum* containing 500 µg/mL phlorotannin to the basal diet in an in vitro batch fermentation. The authors found that cellulolytic bacteria in the rumen such as *F. succinogenes* were inhibited, while non-cellulolytic bacteria increased in the presence of phlorotannins [66]. Zhou et al. [86] monitored the rumen microbiota after addition of ‘Tasco’ (tested at 1%, 3%, or 5% dry matter and incorporated to total mixed ration basal feed), a commercial *A. nodosum* extract, to an in vivo study using 8 cannulated rams. Overall, rumen total bacteria and archaea were reduced. A reduction in pathogenic shiga-toxin-producing *E. coli* population was also observed, indicating the potential of these extracts against foodborne pathogens. In both of the above studies examining the effects of phlorotannins on the rumen microbiome, measurement of CH_4_ levels was not carried out. Thus, further research is required to elucidate the overall microbial dynamics in comparison with CH_4_ emission when phlorotannins are added to ruminant feed.

### Saponins

Saponins are a large class of phytochemicals found in many terrestrial plants and macroalgae [87] that are traditionally used as soap substitutes given their foaming and emulsifying properties [88]. Their chemical structure can vary significantly between species, and as such a variety of biological activities have been reported from these compounds including antimicrobial, hepatoprotective, and immunostimulatory [89]. Saponins have emerged as potential anti-methanogenic agents which has most commonly been linked to their anti-protozoal activity [90, 91]. Macroalgae have been reported to be a source of saponin compounds, particularly green macroalgae [5]. Mani et al. [92] identified saponins in *Ulva lactuca, Halimeda macroloba, C. linum* and *Chaetomorpha antennina*, with *U. lactuca* having the highest saponin contents of 1.77%. However, to date, some of the most common species investigated for their anti-methanogenic activity include saponins from terrestrial plants, mainly *Yucca schidigera* from Mexico and *Quillaja saponaria* commonly found in Chile [93]. There are variable results on the efficiency of these compounds in terms of CH_4_ abatement. A meta-analysis by Jayanegara et al. [93] examining the anti-methanogenic potential of saponins reported that increasing levels of saponins decreased CH_4_ emissions per unit of substrate concurrent with a shift in VFA production towards propionate rather than acetate. However, these effects are source-dependent and can vary between species.

## In vivo studies

In vivo studies incorporating seaweeds into ruminant feed can offer a more complete perspective on the potential for CH_4_ reduction and overall feasibility of this strategy to mitigate ruminant CH_4_ emissions. While in vitro studies are necessary in preliminary stages to assess the potential CH_4_ reduction capacity of seaweeds, further research into the in vivo feasibility of such a strategy is necessary if the agricultural sector is to consider seaweed incorporation in ruminant feed. Any alteration to animal feed can have a variety of off-target effects on meat/dairy quality or overall animal health besides microbiome manipulation/CH_4_ reduction [94]. Moreover, in vivo studies are also crucial to ascertain the persistence of any anti-methanogenic effect observed during in vitro studies with seaweed in ruminant feed. The in vivo studies currently available incorporating seaweed biomass as an anti-methanogenic agent are summarised in Table 3, with all studies focused on *Asparagopsis* spp. as it is the seaweed species reported to be the most effective in reducing CH_4_ emissions in vitro to date. Roque et al. [94] analysed different dosage rates of *A. taxiformis* from 0 to 0.5% (organic matter intake basis) added to a basal feed of low forage total mixed ration diet on 21 Angus-Hereford beef steers in vivo. The authors reported a maximum CH_4_ reduction of 80% in comparison with the control when *A. taxiformis* was applied at the high rate of 0.5%. At the lower rate of 0.25%, a 69% reduction in CH_4_ was observed. The authors reported no change in consumer taste preferences of the strip steak where *A. taxiformis* had been incorporated, and the CH_4_ reduction reported in the study persisted during the 147 d of the trial. Kinley et al. [36] also investigated the inclusion of *A. taxiformis* on 20 Brahman-Angus steers in vivo, and found that over a 90-day period, steers receiving 0.2% (organic matter basis) *A. taxiformis* had CH_4_ emissions reduced by 98% in comparison with the control. A lower dosage rate of *A. taxiformis*, 0.1%, reduced CH_4_ emissions by 40%. No change to quality of meat produced was detected in the sensory analysis conducted in this study. The authors also reported a weight gain of 42%–53% in the treatment animals of the trial. A 65% reduction in CH_4_ emissions was observed by Stefenoni et al. [16] when *A. taxiformis* was applied at 0.5% (dry matter basis) in an in vivo trial with lactating dairy cows, after an initial in vitro trial showed that *A. taxiformis* at 1% dry matter basis reduced CH_4_ emission by 98%. However, the authors reported reduced efficacy of the treatment over time. In the first half of the trial of 56 d a reduction in CH_4_ emissions was observed in comparison with the control; however, no further CH_4_ reduction was demonstrated at later stages during the trial (after 56 d). The authors measured the concentration of bromoform in *A. taxiformis* over time and reported an 84% decrease in bromoform concentration after 4 months of storage. This decrease in bromoform was exacerbated by light; as the samples exposed to light had 17% less bromoform concentration than samples stored in dark conditions [16]. Li et al. [95] observed a dose dependent response in Merino-cross wethers sheep supplemented with *A. taxiformis* at a variety of inclusion rates ranging from 0 to 3% dry matter basis. 80% CH_4_ mitigation in comparison with the control was observed in the treatment group receiving 3% *A. taxiformis*, which persisted over the total of the 72-day trial period. The authors noted that, while sheep offered ≤ 1% *A. taxiformis* consumed all feed, the sheep offered higher dosage rates of 2% and 3% did not always consume all feed. This is also important to note in terms of the practical logistics of CH_4_ mitigation strategies such as feed additive supplementation, and may vary between animals. In this study total VFA concentration was reduced by the inclusion of *A. taxiformis*, however VFA production appeared to be shifted towards propionate. Changes to ruminal mucosa health were noted in certain sheep offered *A. taxiformis*; discolouration and nodular proliferation were observed, and, in all animals, ruminal acidosis was noted in the rumen mucosa [95].

**Table 3:** In vivo studies incorporating seaweeds as anti-methanogenic agents in ruminants’ feed

Seaweeds	Animal	Basal diet	Dosage rates	Effect on CH_4_ emissions	References
*Asparagopsis taxiformis*	Brahman-Angus cross steers	Total mixed ration containing Rhodes grass hay, barley, grain/mineral/vitamin blend	4 dosage rates: 0, 0.05%, 0.10%, and 0.2% of total feed organic matter	0.1% and 0.2% dosage rates reduced CH_4_ emissions by 40% and 92%, respectively	[36]
*Asparagopsis taxiformis*	Merino-cross wethers sheep	Commercial pelleted ration containing high fibre diet (< 2% organic matter)	5 dosage rates: 0, 0.5%, 1%, 2%, and 3% on organic matter basis	3% dosage rate decreased CH_4_ emissions by 80% compared to control	[95]
*Asparagopsis armata*	One Jersey and one Holstein	Total mixed ration	3 dosage rates: 0, 0.5%, and 1% on organic matter basis	0.5% inclusion rate decreased CH_4_ emission by 26.4% 1% inclusion rate decreased CH_4_ emission by 67.2%	[15]
*Asparagopsis taxiformis*	Angus-Hereford beef steers	3 diets fed over time to achieve low forage total mixed ration diet: - Starter diet (63 d) high forage diet - Transition diet (21 d) medium forage - Finisher diet (63 d) Forage contained varying proportions of alfalfa hay, wheat hay, dry distiller grain, and concentrates	3 dosage rates: 0, 0.25%, and 0.5% on organic matter intake basis	Low forage total mixed ration diet supplemented with 0.25% *A. taxiformis* reduced CH_4_ by 69.8% Low forage total mixed ration diet supplemented with 0.5% *A. taxiformis* reduced CH_4_ by 80%	[94]
*Asparagopsis taxiformis*	Lactating Holstein cows	Total mixed ration	3 dosage rates: 0, 0.25%, and 0.5% on dry matter basis	0.5% *A. taxiformis* decreased CH_4_ emission by 65%	[16]

As research into anti-methanogenic activity from seaweeds in vivo is still in the early stages, it remains to be seen what effects might occur within the animal from this supplementation, and if these effects differ between different animals or different diets. Roque et al. [15] reported a 67.2% reduction in CH_4_ emission in comparison with the control when *Asparagopsis armata* was incorporated into the total mixed ration of dairy cattle at an inclusion level of 1% (dry matter basis); while an inclusion rate of 0.5% resulted in 26.4% CH_4_ reduction. This study did not measure VFA production so comparison of rumen fermentation efficiency cannot be ascertained; however, the authors did report reduced milk yields from cows fed the higher inclusion levels of *A. armata.* Cows receiving the lower level of *A. armata* inclusion did not show any difference in milk yields compared to the control. The study also found no difference between milk produced from cows supplemented with *A. armata* and the control with no supplementation. These in vivo studies indicate the potential for CH_4_ mitigation from seaweed incorporation into ruminant feed in practical settings, with CH_4_ reduction of up to 80% observed [94]. For the adoption of this CH_4_ mitigation strategy by industry to be successful, a variety of factors require further research; incorporation of seaweed into ruminant feed must not adversely affect animal health or productivity, or overall ruminant fermentation efficiency.

## Effects of seaweed on the rumen microbiome

Studies analysing effects of seaweed as CH_4_-mitigation agents on the rumen microbiome are crucial to the understanding of the complex dynamics that can occur when any aspect of the rumen microbiome is altered. The main studies investing effects on the rumen microbiome with the addition of seaweed as a feed additive are summarised in Table 4.

**Table 4:** Studies investigating effects on rumen microbiomes after addition of seaweeds/bioactive compounds as feed additives

Seaweeds/ compounds added	Animal	Type of study	Basal diet	Dosage rate	Effects on microbiome	Effect on CH_4_ emissions	References
Bromochloromethane	Murciano-Granadina lactating goats	In vivo: BCM administered 2 equal doses per day from parturition to 2 weeks postweaning	Alfalfa hay with 600 g/d concentrates	0.3 g of BCM/100 kg body weight	No effect on overall abundance of microbial populations	33% reduction in CH_4_	[29]
Bromochloromethane	Brahman-crossbred steers	In vivo: 97 d trial- for first 63 d 100 g cotton seed meal was added to basal diet and for 34 d BCM added to the cotton seed meal	Rhodes grass and grain pellets	0.3 g of BCM/100 kg body weight	Decrease in *Methanobrevivacter* spp. observed when treated with BCM 34% average decrease in methanogenic archaea	30% reduction in CH_4_ emissions	[31]
Bromochloromethane	Non-lactating Friesian-Holstein cattle	In vitro: Batch and continuous fermentation tested	Batch: hay Continuous: 20 g/d hay	Batch: 5 µmol/L and 10 µmol/L tested Continuous: 5 µmol/L	Batch fermentation: 48% decrease in *Ruminococcus flavefaciens*, 68% increase in *Fibrobacter succinogenes* and 30% increase in ruminal fungi Continuous fermentation: significant decrease in *Ruminococcus flavefaciens* and methanogens, no change to *Fibrobacter succinogenes* population, increase in rumen fungi	Batch: 89%–94% reduction of CH_4_ at both concentrations tested Continuous fermentation resulted in 85%–90% CH_4_ reduction	[75]
*Asparagopsis taxiformis* Bromoform	Brahman steers cattle	In vitro: batch fermentation	Rhodes grass	2% of organic matter 2 concentrations tested: 1µmol/L and 5µmol/L	Decrease in CH_4_ production correlated with a decrease in relative abundance of methanogens; particularly Methanobacteriales, Methanomassiliicoccales, Methanomicrobiales.	> 99% reduction compared to basal substrate only control Addition of 1 µmol/L reduced CH_4_ by 77% and 5 µmol/L reduced CH_4_ by > 99% compared to basal substrate-only control	[13]
Bromochloromethane	Japanese native (Shiba) goats	In vivo: animals sequentially adapted to low, medium, and high doses of BCM	50% timothy grass 50% concentrates	Low dose: 0.5 g/100 kg live weight Medium dose: 2 g/100 kg live weight High dose: 5 g/100 kg live weight	Decrease in abundance of methanogens and fungi, and decrease in *Ruminococcus albus* Increases in *Prevotella* spp. and *Fibrobacter succinogenes* No change to *Ruminococcus flavefaciens* or protozoa	Low dose: 5% reduction in CH_4_ Medium dose: 71% reduction in CH_4_ High dose: 91% reduction in CH_4_	[76]
*Asparagopsis taxiformis*	Angus-Hereford beef steers	In vitro: RUSITEC	Super basic ration containing 70% alfalfa pellets, 15% rolled corn, 15% dried distillers grains	5% w/w	Decrease in relative abundance of methanogens compared to the control; decrease was significant when averaged over the course of the experiment	95% reduction in CH_4_ formation	[45]
Phlorotannins extracted from *Ascophyllum nodosum*	Steers	In vitro: batch fermentation	Mixed forage (50:25:25 ground barley silage:alfalfa hay:grass hay)	500 µg/mL phlorotannin extract Extract determined to contain 220 mg phlorotannin/g dry matter	At 500 µg/mL phlorotannin addition, growth of *Fibrobacter succinogenes* reduced by 78% at 6 h, 83% at 12 h, and 65% at 24 h No effect on *Ruminococcus flavefaciens* *Ruminobacter amylophilus, Prevotella bryantii, Selenomonas ruminantium* were increased at 12 and 24 h	Not analysed in this study	[66]
*Undaria pinnatifida* *Sargassum fusiforme* *Sargassum fulvellum*	Non-lactating Hanwoo cows	In vitro batch fermentation	300 mg timothy hay 200 mg corn grain	0.25 mg/mL	*Sargassum fusiforme* increased the overall abundance of total bacteria, ciliate protozoa, fungi, methanogenic archaea, *Fibrobacter succinogenes.* Relative proportions of *Butyrivibrio fibrisolvens, Butyrivibrio proteoclasticus*, and *Prevotella ruminicola* decreased with addition of seaweeds	*Undaria pinnatifida* reduced CH_4_ emission by 26.8% at 12 h and 21.3% at 24 h *Sargassum fusiforme* reduced CH_4_ emission by 23.4% at 12 h and 24.4% at 24 h *Sargassum fulvellum* reduced CH_4_ emission by 26.3% at 12 h and 24.6% at 24 h	[50]
*Ecklonia stolonifera* *Eisenia bicyclis* *Sargassum fulvellum* *Undaria pinnatifida* *Sargassum fusiforme*	Holstein cows	In vitro batch fermentation	Timothy grass	5% dry matter basis	At 12 and 24 h, the abundance of methanogenic archaea decreased in the presence of *Ecklonia stolonifera, Eisenia bicyclis* and *Sargassum fulvellum* At 12 and 24 h, the abundance of methanogenic archaea increased in the presence of *Undaria pinnatifida* and *Sargassum fusiforme*	For all seaweeds except *Sargassum fusiforme*, CH_4_ emission increased at 6 and 24 h incubation After 48 h, CH_4_ was reduced by: 36.1% for *Ecklonia stolonifera* 32.4% for *Eisenia bicyclis* 10.4% for *Sargassum fulvellum* 26.7% for *Undaria pinnatifida* 13.9% for *Sargassum fusiforme*	[51]

Machado et al. [13] examined changes to the rumen microbiome when *A. taxiformis* at 2% (organic matter basis) and bromoform at 5 µmol/L were added to Rhodes grass basal feed in vitro, using rumen inoculum from Brahman steers cattle. Both treatments reduced CH_4_ emission by > 99% in comparison with the control. The authors found that both *A. taxiformis* biomass and bromoform reduced the abundance of the 3 main methanogens in ruminants namely Methanobacteriales, Methanomassiliicoccales and Methanomicrobiales. Roque et al. [45] also reported a decrease in relative abundance of methanogens when a basal feed was supplemented with 5% w/w *A. taxiformis* in an in vitro trial using RUSITEC apparatus and rumen inoculum from Angus-Hereford beef steers. However, this decrease occurred after 72 h, later than the reduction in CH_4_ that was demonstrated after 12 h of treatment (95% reduction in CH_4_ in comparison with the control). The authors suggest that the addition of *A taxiformis* can result in a near-immediate effect on methanogen function, however alterations to the rumen microbial populations can take longer to occur.

As well as the dominant species investigated for CH_4_ mitigation, certain studies have examined brown seaweeds and associated extracts for their effects on microbiome composition. Choi et al. [50] reported CH_4_ reduction of between 21% and 26% in comparison with the control from treatments with seaweed, during an in vitro study using rumen inoculum from non-lactating Hanwoo cows. Extracts of the brown seaweeds *U. pinnatifida, S. fusiforme, and S. fulvellum* were applied at 0.25 mg/mL to a basal feed of timothy hay and corn grain. Addition of *S. fusiforme* increased the overall abundance of total bacteria, ciliate protozoa, fungi, and methanogenic archaea compared to the control; while the addition of *U. pinnatifida* and *S. fulvellum* reduced the abundance of ciliate protozoa and fungi significantly, and neither species decreased the abundance of methanogenic archaea. Certain fibrolytic bacteria populations, including *F. succinogenes* and *Ruminococcus flavefaciens*, were increased by the addition of *S. fusiforme* and *S. fulvellum.* A ruminal fermentation shift towards production of propionate rather than acetate was also observed, presumably due to manipulation of the H_2_ sink after CH_4_ reduction. While CH_4_ reduction was observed in this study, significant reduction in methanogen abundance was not observed, indicating the complex inter-dynamics that can occur within the rumen microbiome that are still not fully understood. The same group analysed 5 species of brown seaweed for similar CH_4_ mitigation and rumen microbiome effects in vitro using rumen inoculum from Holstein cows. *Ecklonia stolonifera*, *Eisenia bicyclis*, *S. fulvellum*, *Undaria pinnatifida*, *S. fusiforme* were all applied at a 5% dry matter basis to a basal diet of timothy grass [51]. After 48 h incubation CH_4_ reduction from between 10% and 36% was observed in comparison with the control. At 12 and 24 h, the abundance of methanogenic archaea, decreased in the presence of *E. stolonifera, E. bicyclis* and *S. fulvellum*, and increased in the presence of *U. pinnatifida* and *S. fusiforme*. Furthermore, the addition of seaweed decreased the abundance of certain cellulolytic bacteria at 24 h including *R. flavefaciens* and *Ruminococcus albus* (Gram-positive), while other cellulolytic bacteria, such as *F. succinogenes* (Gram-negative), experienced increased abundance. The authors noted the presence of phlorotannins in brown seaweeds, which have reported antimicrobial effects particularly in Gram-positive bacteria [96]. As methanogens act in concert with cellulolytic bacteria, such as *R. flavefaciens* and *R. albus*, the decrease in abundance of these bacterial species may contribute to the CH_4_ reduction observed in study. Wang et al. [66] reported manipulation of the rumen microbiome after in vitro supplementation with phlorotannin extracted from the brown seaweed *A. nodosum.* Phlorotannins applied at 500 µg/mL to a basal diet of mixed forage (50:25:25 ground barley silage:alfalfa hay:grass hay) reduced growth of *F. succinogenes* by up to 83%. *R. albus* was reduced at 24 h only and *R. flavefaciens* remained unaffected. This study did not measure CH_4_ output so correlations to CH_4_ mitigation cannot be carried out.

Certain studies applied halogenated compounds in isolation, mainly BCM, for their potential to manipulate the rumen microbiome. Goel et al. [75] observed CH_4_ reductions of 85%–94% in comparison with the control throughout an in vitro study where BCM was applied (at 5 µmol/L) in either batch or continuous fermentation using rumen inoculum from non-lactating Friesian-Holstein cows. During batch fermentation, a 48% decrease in *R. flavefaciens*, 68% increase in *F. succinogenes* and 30% increase in rumen fungi was observed. During continuous fermentation, decreases in *R. flavefaciens* and overall methanogens were observed, with no change to *F. succinogenes* populations, and an increase in rumen fungi. These results were corroborated by Mitsumori et al. [76]. BCM was added (at concentrations of 0.5, 2, and 5 g/100 kg live weight) to basal feed of Shiba goats in vivo, and the authors reported reduced overall rumen methanogen abundance, increased *F. succinogenes*, and appeared to exert no effect on *R. flavefaciens.* Denman et al. [31] also reported decreases in overall methanogenic archaea when BCM was included at 0.3 g/100 kg body weight in a 97-day in vivo trial using 6 Brahman-crossbred steers, concurrent with a 30% observed reduction in CH_4_ emissions in comparison with the control. The authors reported an average 34% decrease in overall methanogen abundance, and a decrease in incidence of *Methanobrevibacter* spp. BCM treatment also resulted in more diverse populations of methanogens, with the main orders represented being Methanococcales, Methanomicrobiales and Methanosacinales. Similar to Roque et al. [45], Denman et al. [31] reported an immediate effect on CH_4_ production when BCM was administered with CH_4_ reduction of 59% within 2 h of bromochloromethane addition; however, methanogen populations only began to decrease after 8 h. The authors suggested that methanogenesis inhibition results in reductions in organism growth, which will take some time to appreciably decrease, while the inhibition of the enzymatic pathways in methanogenesis would result in a more immediate reduction in actual CH_4_ emissions. Abecia et al. [29] did not report any decrease in methanogen populations during an in vivo trial with Murciano-Granadina lactating goats (basal feed of alfalfa hay with 600 g/d concentrates) supplemented with BCM (at 0.3 g/100 kg live weight), resulting in 33% reduction in CH_4_. The authors suggest that methanogen communities can take varying time periods to adapt to any alterations to basal diet, as was reported by Williams et al. [97], and support the hypothesis that the internal population dynamics of methanogens, rather than their overall abundance, is crucial to determine CH_4_ emissions. Roque et al. [45] also observed increased β-diversity in ruminant microbiomes with reduced CH_4_ emission that had been treated with *A. taxiformis*. As the rumen microbiome is a complex organ with multi-dynamics between microbial communities and intra-dynamics within the same community, i.e. methanogen populations, it is necessary for the whole microbiome to be examined (for example via sequencing studies) when any alterations are made via feed additives. Furthermore, when considering the potential of feed additives research must also examine the efficiency of rumen fermentation parameters, the overall health of the animal, and animal productivity.

## Future perspectives, potential risks and challenges

Despite significant CH_4_-mitigation potential, with reductions of approximately 99% reached by the addition of certain seaweed species into ruminants’ feed [13, 16], there remain various challenges and gaps in the knowledge which must be developed and researched further before this strategy may be considered a widespread feasible method of CH_4_-mitigation in practical terms on farms [23].

Further research, both in vitro and in vivo, analysing the ruminant microbiome must be carried out to standardise the seaweed species, dosage, and processing steps to reduce CH_4_ effectively in different animal species and under different farming practices. Varying results regarding CH_4_ abatement are reported in this review, with certain studies reporting complete CH_4_ inhibition [39, 71], moderate CH_4_ reduction of 24%–50% [14, 42, 48] and no effect on CH_4_ emissions [18, 19, 49]. The greatest success has been reported from *A. taxiformi*s, but further research is also necessary into the variety of other species of red, brown, and green seaweeds mentioned in this review to ascertain whether these other species will be suitable for this purpose. The range of dosage rates tested in studies thus far is expansive, from 0.2% whole biomass to 25%, often with dose-dependent responses being observed towards CH_4_ mitigation [40, 95]. The dosage rates for effective CH_4_ reduction may vary between species, and even within the same species depending on the concentration of the bioactive compound of interest (e.g., bromoform), which itself can vary depending on a wide variety of biotic and abiotic factors. Inclusion dosages of > 15% have been reported to have adverse effects on palatability of feed and dry matter intake by the animals [20]. More studies are required to fully understand the relationships between inclusion of seaweed in feed and overall fermentation efficiency, microbiome manipulation, animal digestive health and organoleptic properties of resultant animal meat and dairy products. As mentioned in this review, certain studies report adverse effects on VFA production, generation of H_2_, and in one instance mucosal inflammation in animals after incorporating seaweed in basal feed [48, 76, 95]. Furthermore, any manipulation of the rumen microbiome may cause off-target effects that are as of yet poorly understood, particularly the potential generation of microbiological niches due to depletion of certain microbial communities in the rumen has not yet been established. Further in vivo and microbiome studies in particular are required to ensure that macroalgae addition to basal feed will not negatively affect overall animal health or performance, and animal derived products.

Of particular relevance to strategies implementing *A. taxiformis* as a CH_4_-mitigating agent are the associated toxicology concerns related to bromoform, the primary bioactive compound in this species which appears to inhibit methanogenesis, which has been identified as a carcinogen and ozone-depleting substance [27]. A limit of 80 µg/L bromoform in drinking water has been set by the United States Environmental Protection Agency (US EPA) [98], and the World Health Organisation (WHO) has established a bromoform standard in drinking water of 100 µg/L [99]. A variety of studies have examined the potential for residual bromoform to be present in animal tissues and/or dairy products. It has been reported that bromoform does not appear to accumulate in animal tissue [26, 36], but can appear at low levels in milk. Roque et al. [15] found that milk produced by cows fed *A. taxiformis* at either 0.5% or 1% organic matter contained bromoform at 0.11–0.15 µg/L, which is > 500 times lower than the EPA standard and was not found to be different from the control. Muizelaar et al. [26] reported detection of bromoform at levels as high as 35 µg/L in animals fed *A. taxiformis* at a high level (333 g dry matter), which was undetectable after 17 d. However, the authors noted that animals often refused feed supplemented with *A. taxiformis* and that the trial was terminated early due to poor animal health. Toxicology studies of bromoform reported renal toxicity and hepatotoxicity in rats at 289 mg/kg/d [100], a dosage which is 100–1,000 times higher than average dosage rates used when applying *A. taxiformis.* Bioavailability studies have reported a bromoform half-life of 0.8 h in rats and 8 h in mice [101]. Nevertheless, future studies investigating feed supplementation with *A. taxiformis* should monitor bromoform levels in animal tissues, milk, and excrement, to ensure compliance with regulatory standards.

As well as bromoform, concerns have been noted regarding the potential for iodine and other heavy metal accumulation, such as Cd and Hg, and As, resulting from ruminant feed supplementation with seaweeds. The European Food Safety Authority (EFSA) has published a recommended maximum dosage of iodine in milk to be 500 mg/L [102]. Antaya et al. [103] reported increase in iodine levels from milk of Jersey cows fed *A. nodosum*, with a high dosage of *A. nodosum* (170 g/d) resulting in 1,370 mg/L iodine in milk. It has been suggested that this increase in iodine content in milk as a result of seaweed could be incorporated into dietary strategies to fortify milk products in populations with iodine deficiency [23]; however, this would require further investigation and regulation [9]. The North Ronaldsay sheep in Orkney, whose feed consists of > 90% seaweed [104], have been reported to accumulate high levels of arsenic, a metal that has been linked to several health issues in these animals, including dental disease [105]. In 2018, the European Commission issued a recommendation to monitor the levels of As, I, Pb, Cd, and Hg in macroalgae food and feed products, including feed additives [25]. Such regulation will greatly affect the potential for widespread adoption of seaweed as a ruminant feed additive for CH_4_ mitigation. Recently, Noriega-Fernández et al. [106] reported that processing techniques can reduce the levels of these elements in *Laminaria hyperborea;* a combination of ultrasound and EDTA treatment resulted in a 32% reduction in arsenic, 52% reduction in cadmium, and 31% reduction in iodine present in this seaweed.

The cultivation of macroalgal biomass at sufficient levels to implement as a feed additive to mitigate CH_4_ emissions represents a future industrial challenge, reviewed previously by Nilsson and Martin [107] and Cerca et al. [108]. McCauley et al. [20] presents an example scenario whereby feeding a dairy farm herd of 350 cows macroalgal biomass of 0.5% dry matter per day would require ~ 265 kg fresh algae each day, considering a moisture loss of 90% from the drying process. Worldwide, significant portions of the macroalgal industry are sourced from harvested natural biomass. This reduces the capital expenditure required for seaweed producing companies; however, it can have deleterious effects on the marine environment and ecosystems and contribute to biodiversity loss. Seaweed farms are also operated both on land and in the marine environment with opportunities and challenges associated with each. Offshore seaweed cultivation farms do not require investment in optimising cultivation conditions as on-land cultivation schemes do, they do not compete for land use with food for human consumption, and can benefit the marine environment via carbon sequestration and providing habitats for marine organisms [23]. However, with increasingly scaled-up cultivation any potential concerns for heavy metal accumulation will also increase, as well as the unknown ramifications of significantly shifting existing balanced ecosystems in marine environments via the addition of seaweed. Land-based seaweed cultivation systems require extensive investment to ensure optimal cultivation conditions. However, there is an opportunity to cultivate seaweeds in integrated multi-trophic aquaculture systems with other marine life, which can utilise recirculating water or could even make use of industrial waste streams to contribute to a circular economic model.

As well as upstream generation of sufficient biomass to satisfy potential for CH_4_ mitigation, processing of harvested seaweed, regardless of source, will require significant optimisation to ensure success of scaled-up industries. Due to the high water content in macroalgae and the potential for the biomass to decay quickly, various post-harvesting steps including drying need to be carried out in a short time frame, often hours after harvesting [108]. Recently, Magnusson et al. [27] developed a stable bromoform product from fresh *A. taxiformis* in an oil emulsion; which was shelf-stable for 12 weeks. This strategy avoids the need for drying macroalgal biomass, which is one of the main bottlenecks in post-harvesting efficiency [108]. Transport, storage, and preservation are also post-harvesting steps that must be considered when scaling up macroalgal production for CH_4_-mitigation purposes, complicated by the seasonality of macroalgal aquaculture which can mean extensive capital expenditure on machinery that are only used for a certain number of months in a year. Life-cycle assessments and technoeconomic analysis must be carried out to ascertain the feasibility of industrial scale macroalgal aquaculture, with bioeconomic modelling approaches recommended [5]. Nilsson and Martin [107] carried out an exploratory environmental assessment on large-scale land-based cultivation of *A. taxiformis* for reduction of enteric CH_4_, and reported increased water recycling, sustainable heat sources, and source of salt used to be the most dominant factors in determining the overall environmental sustainability and feasibility of this system. Despite the potential challenges, there is increasing global interest in industrial macroalgal production and commercialisation, particularly in countries such as New Zealand, Australia, and certain European countries, such as Spain and France; supported by partnerships between universities and industry and government grant and research schemes [20]. The potential for macroalgal cultivation to contribute to a biorefinery system, whereby multiple revenue streams are generated from a natural capital or single biomass, has increased global interest in a variety of stakeholders, releasing more private and public investments for exploitation of this biomass [109].

## Conclusions

Mitigation of ruminant CH_4_ emissions via the incorporation of seaweeds in basal feed has potential to be a successful strategy to reduce overall agricultural CH_4_ emissions. While efficacy of CH_4_ reduction varies between studies, the most successful results to date have been reported from the red seaweed *Asparagopsis taxiformis* at dosage rates of ~ 2% organic matter, attributed to the halogenated compound bromoform which disrupts methanogenesis. These results have been accompanied by observed reductions in methanogen abundance in the rumen microbiome, and manipulations of rumen bacteria and protozoa. Further research is required to optimise CH_4_ mitigation strategies with *A. taxiformis*, and to determine if other seaweed species can reduce CH_4_ emissions with the same efficacy in vitro and in vivo. Furthermore, microbiome studies should examine overall effects on the ruminant microbiome following treatment with seaweeds or seaweed bioactives. Future challenges regarding industrial adoption of seaweed-based CH_4_ mitigation strategies include standardisation of dosage (both of whole seaweed and bioactive compounds within) and effects on animal health and animal products, toxicology of certain compounds within and accumulated by seaweeds, and the feasibility of large scale cultivation of seaweed biomass.

## Data Availability

All data is available in the current version of the manuscript.

## Abbreviations

3NOP: 3-nitrooxypropanol
BCM: Bromochloromethane
CH_3_: Methyl group
CH_4_: Methane
CO_2_: Carbon dioxide
CoA: Coenzyme A
CoM: Coenzyme M
DW: Dry weight
EDTA: Ethylenediaminetetraacetic acid
EFSA: The European Food Safety Authority
GHG: Greenhouse gas
GWP: Global warming potential
H_2_: Hydrogen
IPCC: Intergovernmental Panel on Climate Change
Mcr: Methyl-coenzyme M reductase
PUFA: Poly unsaturated fatty acids
RUSITEC: Rumen simulation technique
US EPA: United States Environmental Protection Agency
VFA: Volatile fatty acids
WHO: World Health Organisation
